# Effect of supplementation with vitamins D3 and K2 on undercarboxylated osteocalcin and insulin serum levels in patients with type 2 diabetes mellitus: a randomized, double-blind, clinical trial

**DOI:** 10.1186/s13098-020-00580-w

**Published:** 2020-08-18

**Authors:** J. I. Aguayo-Ruiz, T. A. García-Cobián, S. Pascoe-González, S. Sánchez-Enríquez, I. M. Llamas-Covarrubias, T. García-Iglesias, A. López-Quintero, M. A. Llamas-Covarrubias, J. Trujillo-Quiroz, E. A. Rivera-Leon

**Affiliations:** 1 Pharmacology, Health Sciences University Center (CUCS), Universidad de Guadalajara (UdeG), 44350 Guadalajara, Jalisco Mexico; 2 Department of Molecular Biology and Genomics, Health Sciences University Center (CUCS), Universidad de Guadalajara (UdeG), 44350 Guadalajara, Jalisco Mexico; 3 Department of Physiology, Health Sciences University Center (CUCS), Universidad de Guadalajara (UdeG), 44350 Guadalajara, Jalisco Mexico; 4 Department of Clinics, Altos University Center (CuAltos), Universidad de Guadalajara (UdeG), 47620 Tepatitlán de Morelos, Jalisco Mexico

**Keywords:** Vitamin D, Vitamin K, T2DM, Osteocalcin, Insulin

## Abstract

**Background:**

Patients with type 2 diabetes mellitus (T2DM) are characterized by chronic hyperglycemia as a consequence of decreased insulin sensitivity, which contributes to bone demineralization and could also be related to changes in serum levels of osteocalcin and insulin, particularly when coupled with a deficiency in the daily consumption of vitamins D3 and K2. The objective of this study was to evaluate the effect of vitamin D3 and vitamin K2 supplements alone or in combination on osteocalcin levels and metabolic parameters in patients with T2DM.

**Methods:**

A double-blind, randomized clinical trial was carried out in 40 patients aged between 30 and 70 years old for 3 months. Clinical and laboratory assessment was carried out at the beginning and at the end of the treatment. The patients were divided into three groups: (a) 1000 IU vitamin D3 + a calcinated magnesium placebo (n = 16), (b) 100 µg of Vitamin K2 + a calcinated magnesium placebo (n = 12), and (c) 1000 IU vitamin D3 + 100 µg vitamin K2 (n = 12).

**Results:**

After treatment in the total studied population, a significant decrease in glycemia (p = 0.001), HOMA-IR (Homeostatic model assessment-insulin resistance) (p = 0.040), percentage of pancreatic beta cells (p < 0.001), uOC/cOC index and diastolic blood pressure (p = 0.030) were observed; in vitamin D3 group, differences in serum undercarboxylated osteocalcin (p = 0.026), undercarboxylated to carboxylated osteocalcin index (uOC/cOC) (p = 0.039) glucose (p < 0.001) and  % of functional pancreatic beta cells (p < 0.001) were demonstrated. In vitamin K2 group a significant decrease in glycemia (p = 0.002), HOMA-IR (p = 0.041), percentage of pancreatic beta cells (p = 0.002), and in cOC (p = 0.041) were observed, conversely cOC concentration was found high. Finally, in the vitamins D3 + K2 a significant decrease in glycemia (p = 0.002), percentage of pancreatic beta cells (p = 0.004), and in the uOC/cOC index (p = 0.023) were observed.

**Conclusion:**

Individual or combined supplementation with vitamins D3 and K2 significantly decreases the glucose levels and  % of functional pancreatic beta cells, while D3 and D3 + K2 treatments also induced a reduction in the uOC/cOC index. Only in the group with vitamin D3 supplementation, it was observed a reduction in undercarboxylated osteocalcin while vitamin K2 increased the carboxylated osteocalcin levels.

*Trial registration* NCT04041492

## Background

Diabetes mellitus (DM) is a group of metabolic diseases characterized by hyperglycemia as a result of defects in the secretion or action of insulin or both. Chronic hyperglycemia in diabetes has been associated in the long term with damage, dysfunction or failure of certain organs, specifically the eyes, kidneys, nerves, heart, and blood vessels [1].

In 2012, approximately 350 million people around the world suffered from some type of DM, with an estimated reduction in lifespan between 5 and 10 years [2, 3].

The therapeutic strategy is somewhat complicated on occasions by the requirement of various medications such as oral antidiabetic agents and insulin, with the goal of preventing acute decompensation and preventing or limiting long term comorbidity, improving quality of life and decreasing mortality rates [4].

Osteoporosis and T2DM are pathologies that have shown association with an increased risk of stress fractures [5–7]. Hyperglycemia affects the adequate function of osteoblasts which is mediated by their sensitivity to an acidic environment produced by lactate, causing alterations in the formation of collagen fibers, in secondary mineralization and in the formation of the protein-rich extracellular matrix [8].

Vitamin D3 (VD3) deficiency decreases the availability of Ca^2+^ and leads to bone structural defects, similar to those which occur in the catabolic state that leads to osteoporosis [6]. Administration of VD3 promotes intestinal absorption of calcium and phosphorus, increases renal tubular reabsorption of calcium, increases the expression of calbindin and accelerates the transport of calcium in the distal convoluted tubule of the nephron [9]. Mini-remodeling of the bone suffers a considerable increase, augmenting mineralization, and limiting the accumulation of microscopic structural damage [10, 11]. Vitamin K2 has been described as a key element in the preservation of bone health, given that it plays an integral role in the gamma-carboxylation of osteocalcin, and promotes the integration of calcium into the bone [12]. Supplementation of vitamin K2 has been shown to reduce the risk of diabetes development, that just 10 µg/day of K2 decreases diabetes risk by 7%. The mechanism by which K2 may act in doing so is beginning to be elucidated [13]. Calcium, vitamin D3, and K2 are supplements used as adjuvants to achieve better bone health [14].

Osteocalcin is a 49 amino acid peptide synthesized in osteoblasts and by post-translational modification the fully carboxylated (cOC) and partially carboxylated (uOC) forms are produced. Carboxylated osteocalcin has a high affinity for the bone mineral matrix [15]. Undercarboxylated osteocalcin has metabolic effects within the pancreas and adipose tissue, increasing the synthesis and secretion of insulin in beta cells and stimulating the release of adiponectin from adipose tissue. Thus, improving the sensitivity to insulin and improving glucose metabolism as well [16]. The current study analyzed the association between Vitamins D3 and K2 supplementation in T2DM patients and serum concentrations of carboxylated and undercarboxylated osteocalcin.

## Methods

A controlled, double-blind, randomized study was conducted in a group of 40 patients which underwent the following interventions for 3 months. Once the subjects accepted to participate in the study a physician performed the medical record by structured interviews. This medical record included a questionnaire with sociodemographic data, clinical assessment, anthropometrics as well as lifestyle habits, and drug addiction. All data were uploaded to an Excel^®^ sheet. After proving the inclusion criteria, the included patients had preexisting T2DM, they were from Western Mexico, both genders, between 30 and 75 years. T2DM diagnosis was confirmed according to the American Diabetes Association (ADA) criteria and patients who had at least 5 years since T2DM diagnosis were enrolled. All participants signed an informed consent form, it was proceeded with the random assignment by a closed envelope which contained the information of the corresponding random number. Randomization was carried out by someone outside the research team, this person maintained in custody the pills and treatment assignment codes. Also, this person was in charge of the delivery and reception of the self-reporting compliance diary and counting pills and vials.

The assignment of the patient’s random numbers was carried out by SPSS software. The patients were trained to register all important events in the compliance diary to assure the correct intake, as well as adverse effects on them. This was assessed during the scheduled appointments. Data were kept under strict confidentiality and it was only possible to access them upon finishing the study. The adherence was determined by Test Morosky Green-Levine.

It was a double-blinded study, which was achieved by keeping similarities in appearance, tasting, and smell among treatments randomly assigned and delivered in equal vials, with an identical number of pills in shape, size, color, and consistency for all study groups. In the last visit, it was shown the obtained results during the trial for each patient.

Group 1: Vitamin D3 (Essential Nutrition, Mexico, COFEPRIS authorization 173300201A0652) 1000 IU + Calcined magnesia (placebo), Group 2: Vitamin K2 (Essential Nutrition, Mexico, COFEPRIS authorization 173300201A0652) 100 µg + Calcined Magnesia (Placebo), Group 3: Vitamins D3 1000 IU + K2 100 µg.

### Serum levels osteocalcin, insulin and biochemical analysis

3 mL of peripheral blood was drawn from each participant’s forearm using a punzocat. Undercarboxylated osteocalcin levels were determined using the following kit: Undercarboxylated osteocalcin Glu-Oc enzyme-linked immunoSorbent assay (ELISA) Kit, MK-118 (Takara, USA), Carboxylated osteocalcin Human Osteocalcin Quantikine ELISA DSTCN0 R&D System (Minneapolis, USA), Insulin, Insulin Test System ELISA Kit, 2425-300 (Monobind, USA). All measurements were conducted following the manufacturer instructions. Glucose, cholesterol, c-VLDL and triglycerides serum quantifications were performed using the clinical chemistry analyzer, model ERBA XL 200.

Evaluation of the homeostatic model assessment-insulin resistance (HOMA-IR) was calculated by using the HOMA-IR and HOMA-^®^ or F^®^C % [17] equations for insulin resistance (IR) and the percentage of functional pancreatic beta cells, respectively. HOMA-IR: [fasting glucose (mg/dL)  ×  fasting insulin (µU/mL)]/405; HOMA-B: [fasting plasma insulin (µU/mL) × 360]/[fasting plasma glucose (mg/dL) − 63].

### Sample size

Sample size was calculated using the statistical formula for the comparison of two means (a), considering serum levels of undercarboxylated osteocalcin in the general population as the outcome variable, 95% statistical confidence, 80% statistical power and statistical significance of p ≤ 0.05. The sample size calculated was 12 participants, 20% more was added to account for possible losses, obtaining a final sample size of 15 patients per group.

The statistical formula for the comparison of two means

1 $$N = \frac{{\left( {r + 1} \right)\left( {Z + \frac{\alpha }{2} Z 1 - \beta } \right)^{2} \sigma^{2} }}{{rd^{2} }}$$

### Statistical analysis

The Shapiro–Wilk normality test was conducted, qualitative variables are expressed as frequencies and percentages, while quantitative variables are expressed as mean and SD. Inter-group differences were analyzed using the Kruskal–Wallis test, while intra-group differences were analyzed with the Wilcoxon test. All data were analyzed using IBM^®^ SPSS^®^ Statistics 21 for Windows. A p value < 0.05 was considered statistically significant.

### Ethics committee approval

The study was approved by the ethics and research committee of the Health Sciences University Center, University of Guadalajara, with the following registry number: F-2017-1702-14 and in Clinical Trials: NCT04041492.

## Results

Total population was 40 patients distributed into 3 different groups: supplementation with Vitamin D3 (n = 16), supplementation with Vitamin K2 (n = 12) and supplementation with Vitamin D3 + K2 (n = 12).

The baseline characteristics of the studied population are shown in Table 1. The average years of education were 11.4 ± 4.9, with an average time since T2DM diagnosis of 10.4 ± 4.8 years. Metformin was used as treatment in 85% of patients, while the rest followed another antidiabetic treatment plan. Postmenopausal status was observed in 45% of participating women, 57.7% of the population actively consumed alcohol, 17.5% smoked and 60% had a sedentary lifestyle with no differences among groups.

**Table 1:** Pre- and post-treatment characteristics among study groups

Variable		Treatment	Pre-treatment		Post-treatment	
			Mean ± SD	p value	Mean ± SD	p value
Age (years)^c^	D		55.31 ± 8.85	0.889^a^	NA	
	K		55.42 ± 12.62			
	D+K		57.08 ± 9.59			
Alcohol consumption^d^	D		62.5 (10)	0.405^b^	NA	
	K		41.7 (5)			
	D+K		66.7 (8)			
Smoking^d^	D		6.3 (1)	0.311^b^	NA	
	K		25 (3)			
	D+K		25 (3)			
Physical activity^d^	D		50 (8)	0.574^b^	NA	
	K		33.3 (4)			
	D+K		33.3 (4)			
Weight (kg)^c^	D		82.09 ± 18.16	0.670^a^	80.56 ± 16.58	0.775^a^
	K		76.08 ± 20.86		75.80 ± 20.26	
	D+K		77.62 ± 15.64		78.58 ± 15.31	
BMI (kg/m^2^)^c^	D		30.23 ± 6.25		29.71 ± 5.93	0.770^a^
	K		28.49 ± 7.44		28.38 ± 7.14	
	D+K		27.91 ± 4.55	0.586^a^	28.26 ± 4.42	
Muscle (%)^c^	D		51.38 ± 9.28	0.337^a^	51.29 ± 8.45	0.370^a^
	K		46.01 ± 9.27		46.18 ± 10.00	
	D+K		50.48 ± 10.88		50.79 ± 11.69	
Fat (%)^c^	D		32.34 ± 10.69	0.875^a^	33.62 ± 9.74	0.795^a^
	K		34.38 ± 9.89		34.78 ± 9.61	
	D+K		34.24 ± 14.67		31.98 ± 11.32	
SBP (mmHg)^c^	D		132.25 ± 16.60	0.870^a^	128.38 ± 16.13	0.914^a^
	K		132.33 ± 27.19		130.17 ± 18.33	
	D+K		136.33 ± 23.18		126.92 ± 22.22	
DBP (mmHg)^c^	D		87.81 ± 13.49	0.252^a^	85.31 ± 14.85	0.138^a^
	K		81.17 ± 14.14		78.17 ± 8.79	
	D+K		80.92 ± 8.35		76.67 ± 10.67	
Glucose (mg/dL)^c^	D		132.37 ± 18.71	0.627^a^	117.50 ± 17.50	0.520^a^
	K		144.17 ± 35.76		130.25 ± 29.70	
	D+K		138.25 ± 40.65		123.58 ± 39.05	
Insulin (ng/dL)^c^	D		92.24 ± 50.13	0.603^a^	94.03 ± 49.89	0.363^a^
	K		108.60 ± 54.59		85.17 ± 43.59	
	D+K		114.32 ± 76.68		113.75 ± 55.41	
cOC (ng(dL)^c^	D		0.809 ± 0.726	0.193^a^	0.986 ± 0.626	0.131^a^
	K		0.818 ± 0.567		1.224 ± 1.092	
	D+K		0.441 ± 0.304		0.611 ± 0.278	
uOC (ng/dL)^c^	D		3.326 ± 1.774	0.097^a^	2.525 ± 1.566	0.754^a^
	K		2.534 ± 1.616		2.355 ± 1.236	
	D+K		2.046 ± 1.010		2.136 ± 1.119	
HOMA-IR^c^	D		29.44 ± 14.99	0.333^a^	26.78 ± 13.30	0.406^a^
	K		41.60 ± 28.22		28.66 ± 19.96	
	D+K		39.06 ± 25.39		35.84 ± 21.12	
%FPβC^c^	D		546.33 ± 427.38	0.776^a^	237.90 ± 89.21	0.301^a^
	K		514.79 ± 204.01		174.21 ± 04.77	
	D+K		622.40 ± 444.25		281.51 ± 87.39	
Cholesterol (mg/dL)^c^	D		195.88 ± 36.73	0.801^a^	198.00 ± 48.66	0.789^a^
	K		197.08 ± 41.52		206.08 ± 34.18	
	D+K		204.50 ± 25.94		194.92 ± 36.24	
Triglycerides (mg/dL)^c^	D		202.50 ± 142.56	0.856^a^	221.75 ± 115.46	0.155^a^
	K		180.67 ± 115.08		188.08 ± 79.55	
	D+K		179.42 ± 106.34		154.83 ± 44.37	
VLDL (mg/dL)^c^	D		40.19 ± 28.78	0.875^a^	44.35 ± 23.09	0.155^a^
	K		36.13 ± 23.02		37.62 ± 15.91	
	D+K		35.88 ± 21.27		30.97 ± 8.87	

*NA* not applicable, *BMI* body mass index, *SBP* systolic blood pressure, *DBP* diastolic blood pressure, *HOMA-IR* homeostatic model assessment-insulin resistance,  *% FPβC* percentage of functional pancreatic beta cells, *VLDL* very low density lipoprotein, *SD* standard deviation

^a^Mean values were compared using ANOVA test

^b^Number of individuals were compared by Chi square test

^c^Data presented as average and standard deviation

^d^Data presented as percentage and number

The main objective of the study was to assess undercarboxylated osteocalcin (uOC) and serum insulin levels after vitamin D3 and K2 supplementation. In this regard, there were no significant differences for uOC nor insulin levels, when the groups were considered as a whole population (Table 2). On the other hand, vitamin D3 supplementation significantly decreased uOC levels while insulin levels remained the same (Table 3). In the case of vitamin K2, there were not found differences in the before mentioned variables (Table 4), and the same occurred with the simultaneous administration of vitamin D3 + K2 (Table 5).

**Table 2:** Pre-treatment vs post-treatment comparison in clinical and biochemical parameters in whole studied population

Variable	Initial n = 40	Final n = 40	p value
Gender			
Female (N %)	24 (60)		–
Male (N %)	16 (40)		–
Age (years)	55.9 ± 10.0		–
BMI (kg/m^2^)	29.0 ± 6.1	28.8 ± 5.8	0.539
uOC (ng/dL)	2.7 ± 1.5	2.3 ± 1.3	0.104
cOC (ng/dL)	0.701 ± 0.591	0.945 ± 0.753	*0.004*
uOC/cOC index	6.0 ± 5.5	3.4 ± 2.2	*0.001*
Insulin (ng/dL)	103.7 ± 59.6	97.2 ± 49.9	0.428
Glucose (mg/dL)	137.6 ± 31.4	123.1 ± 28.7	*0.001*
HOMA-IR	3.5 ± 2.2	3.0 ± 1.7	*0.040*
% FPβC	559 ± 373	231 ± 169	*0.001*
SBP (mmHg)	133 ± 21	128 ± 18	0.082
DBP (mmHg)	83 ± 12	80 ± 12	*0.030*
Cholesterol (mg/dL)	198 ± 34	199 ± 40	0.625
Triglycerides (mg/dL)	189 ± 21	191 ± 90	0.624
VLDL (mg/dL)	38 ± 25	38 ± 18	0.624

*uOC* undercarboxylated osteocalcin, *cOC* carboxilated osteocalcin, *HOMA-IR* homeostatic model assessment-insulin resistance, *SBP* systolic blood pressure, *DBP* diastolic blood pressure, *FPβC* functional pancreatic β cells. Data presented as average and standard deviation. Statistically significant data are shown in italics type (p < 0.05)

**Table 3:** Pre-treatment vs post-treatment comparison of clinical and biochemical parameters in T2DM patients with vitamin D3 supplementation

Variable	Initial N = 16	Final N = 16	p value
Glucose (mg/dL)	138 ± 18	117 ± 17	*0.001*
HOMA-IR	2.9 ± 1.5	2.7 ± 1.3	0.469
%FPβC	546 ± 427	237 ± 189	*0.001*
SBP (mmHg)	132 ± 16	128 ± 16	0.109
DBP (mmHg)	87 ± 13	85 ± 14	0.187
Cholesterol (mg/dL)	195 ± 36	198 ± 48	0.995
Triglycerides (mg/dL)	202 ± 142	221 ± 115	0.469
VLDL (mg/dL)	40 ± 28	44 ± 23	0.469
uOC (ng/dL)	3.3 ± 1.7	2.5 ± 1.5	*0.026*
cOC (ng/dL	0.809 ± 0.726	0.986 ± 0.626	0.156
uOC/cOC index	7.0 ± 7.0	3.1 ± 1.7	*0.039*
Insulin (ng/dL)	92 ± 50	94 ± 49	0.877
Muscle (%)	51 ± 9	51 ± 8	0.552
Fat (%)	32 ± 10	33 ± 9	0.443
BMI (kg/m^2^)	30 ± 6	29 ± 5	0.995
Weight (kg)	82 ± 18	80 ± 16	0.995

*HOMA-IR* homeostatic model assessment-insulin resistance, *% FPβC* percentage of functional pancreatic beta cells, *SBP* systolic blood pressure, *DBP* diastolic blood pressure, *VLDL* very low density lipoprotein, *uOC* undercarboxylated osteocalcin, *cOC* carboxilated osteocalcin, *BMI* body mass index. Data presented in average and standard deviation. Statistically significant data are shown in italics type (p < 0.05)

**Table 4:** Pre-treatment vs post-treatment comparison of clinical and biochemical parameters in T2DM patients with vitamin K2 supplementation

Variable	Initial N = 12	Final N = 12	p value
Glucose (mg/dL)	130 ± 29	114 ± 35	*0.002*
HOMA-IR	4.1 ± 2.8	2.8 ± 1.9	*0.041*
%FPβc	514 ± 204	174 ± 104	*0.002*
SBP (mmHg)	132 ± 27	130 ± 18	0.695
DBP (mmHg)	81 ± 14	78 ± 8	0.116
Cholesterol (mg/dL)	197 ± 41	206 ± 34	0.239
Triglycerides (mg/dL)	180 ± 115	188 ± 79	0.814
VLDL (mg/dL)	36 ± 23	37 ± 15	0.814
uOC (ng/dlL	2.5 ± 1.6	2.3 ± 1.2	0.638
cOC (ng/dL	0.818 ± 0.567	1.2 ± 1.1	*0.041*
uOC/cOC index	4.3 ± 4.2	2.7 ± 1.9	0.182
Insulin (ng/dL)	108 ± 54	85 ± 43	0.117
Muscle (%)	46 ± 9	46 ± 10	0.657
Fat (%)	34 ± 9	34 ± 9	0.875
BMI (kg/m^2^)	28 ± 7	28 ± 7	1.000
Weight (kg)	76 ± 20	75 ± 20	1.000

*HOMA-IR* homeostatic model assessment-insulin resistance, *% FPβC* percentage of functional pancreatic beta cells, *SBP* systolic blood pressure, *DBP* diastolic blood pressure, *VLDL* very low density lipoprotein, *uOC* undercarboxylated osteocalcin, *cOC* carboxilated osteocalcin, *BMI* body mass index. Data presented in average and standard deviation. Statistically significant data are shown in italics type (p < 0.05)

**Table 5:** Pre-treatment vs post-treatment comparison of clinical and biochemical parameters in T2DM patients with vitamin D3 plus K2 supplementation

Variable	Initial n = 12	Final n = 12	p value
Glucose (mg/dL)	138 ± 40	123 ± 39	*0.002*
HOMA-IR	3.9 ± 2.5	3.5 ± 2.1	0.388
%FPβC	622 ± 444	281 ± 187	*0.004*
SBP (mmHg)	136 ± 23	126 ± 22	0.209
DBP (mmHg)	80 ± 8	76 ± 10	0.286
Cholesterol (mg/dL)	204 ± 25	194 ± 36	0.530
Triglycerides (mg/dL)	179 ± 106	154 ± 44	0.937
VLDL (mg/dL)	35 ± 21	30 ± 8	0.937
uOC (ng/dL)	2.0 ± 1.0	2.1 ± 1.1	1.000
cOC (ng/dL	0.441 ± 0.304	0.611 ± 0.278	0.065
uOC/cOC index	6.4 ± 4.2	4.3 ± 2.9	*0.023*
Insulin (ng/dL)	114 ± 76	113 ± 55	0.638
Muscle (%)	50 ± 10	50 ± 11	0.182
Fat (%)	34 ± 14	31 ± 11	0.433
BMI (kg/m^2^)	27 ± 4	28 ± 4	0.050
Weight (kg)	77 ± 15	78 ± 15	0.055

*HOMA-IR* homeostatic model assessment-insulin resistance, % *FPβC* percentage of functional pancreatic beta cells, *SBP* systolic blood pressure, *DBP* diastolic blood pressure, *VLDL* very low density lipoprotein, *uOC* undercarboxylated osteocalcin, *cOC* carboxylated osteocalcin, *BMI* body mass index. Data presented in average and standard deviation. Statistically significant data are shown in italics type (p < 0.05)

Comparative data of secondary analysis between the initial and final determinations for total studied population showed statistically significant decrease for glucose (p < 0.001), HOMA-IR (p = 0.040), diastolic blood pressure (p = 0.030), percentage of functional pancreatic beta cells (p = 0.001), and uOC/cOC index (p = 0.001) while the concentration of cOC was the only significantly increased (p = 0.004) (see Table 2).

At the end of the study no sex differences regarding glucose (p = 0.733), HOMA-IR (p = 0.734), percentage of functional pancreatic beta cells (p = 0.692), systolic blood pressure (p = 0.075) and lipid profile: cholesterol (p = 0.279), triglycerides (p = 0.436) and VLDL (p = 0.436) were observed. However, women showed significant higher levels of diastolic blood pressure (p = 0.025), uOC (p = 0.048) and body fat percentage (p < 0.001), conversely, men had higher percentage of muscle mass (p < 0.001). Data not shown in tables.

After 3 months of treatment, the intragroup analysis of the initial and final data points in the vitamin D3 group showed significant differences regarding decreases in glucose (p = 0.001), percentage of functional pancreatic beta cells (p = 0.001), in the serum levels of uOC (p = 0.026) and the uOC/cOC index (p = 0.039), in the rest of variables no significant changes were observed (see Table 3).

Intervention with vitamin K2, showed significant decrease in plasma levels of glucose (p = 0.002), HOMA-IR (p = 0.041), the percentage of functional pancreatic beta cells (p = 0.002) and significant increase of cOC concentration (p = 0.041) (see Table 4).

The group with administration of Vitamins D3 and K2 combination demonstrated decreases in glucose (p = 0.002), the percentage of functional pancreatic beta cells (p = 0.004) and the uOC/cOC index (p = 0.023). A trend was observed for cOC, BMI and total body weight (p = 0.050 and p = 0.055, respectively) (see Table 5).

An analysis carried out between the vitamin K2 and the vitamin D3 + K2 intervention groups demonstrated statistical significance for percentage of functional pancreatic beta cells with p = 0.038. A greater decrease in the percentage of functional pancreatic beta cells was observed in the groups supplemented with vitamin K2.

Adherence to treatment among groups was as follows: Vitamin D had 100% adherence while vitamin K and D + K groups had 90%. The adherence among groups was compared with the Fisher’s Exact test with no significant differences (p = 0.213). No adverse effects were reported in any of the groups during this study.

## Discussion

The prevalence of T2DM worldwide has been increasing at an alarming rate, with a higher prevalence among the female population (55%) according to studies conducted, similar results were found in the present study, where more than half of the participants were women (60%). Therefore, a special emphasis must be given to this group to prevent complications, especially considering the post-menopausal decrease of hormonal protective factors and the fact that most of the study population included was 55 ± 6 years old. This is relevant since the presentation ages are similar in menopause and in T2DM [18].

There is special attention to maintain health in the T2DM patients through the monitoring of biomarkers and the use of adjunctive/alternative therapies. This has led to the research of osteocalcin and its different biochemical forms (fully carboxylated, partially or undercarboxylated, decarboxylated and total osteocalcin) [19, 20]. Growing evidence maintains the discrepancy over the metabolic implications of OC and importance of the metabolically uOC form, which has been related to regulation of glucose and other parameters, while other authors make emphasis on the different proposed ratios among OC [19, 21–24]. Moreover, vitamin D and K supplementation has shown interesting results on improving metabolic markers [13, 21, 25, 26], in this regard, this study aimed to analyze the effect of supplementation with vitamins D3 and K2 on uOC and insulin serum levels in T2DM patients.

In the basal state, all the included patients showed insulin resistance. After 3 months of treatment, a decrease in the HOMA-IR value was observed in all studied groups, however, only in the total population and in the group with vitamin K2 supplementation this decrease was statistically significant, which can be interpreted as an improvement in the insulin sensitivity (Tables 2 and 4). A significant reduction of HOMA-B at the end of the study in all treatment groups was observed. In literature it is established a wide range of values to consider insulin resistance in adults, typically around 2–2.5 [27–29]. Notwithstanding the discrepancies with the cut-off HOMA-IR-values, the included subjects were characterized by HOMA-IR above 2.5 as expected for T2DM patients. An increased beta cell function coupled with poor insulin sensitivity has been reported for individuals with metabolic syndrome [27], this situation was evidenced at the beginning of the study while a reduction at the end was observed.

Ortega et al. reported that vitamin K deficiency was present in 30.2% of the Spanish population, even though this molecule supports multiple processes that are essential for the proper functioning of the organism, such as coagulation and carboxylation of osteocalcin. It is able to function as a protective factor in bone metabolism and also participates in glucose metabolism and increases insulin sensitivity of patients with T2DM [30]. This was demonstrated in our study with decreases in the HOMA-IR, reflecting an improvement in insulin sensitivity, with a lower amount of circulating insulin, which allows for the reduction of the percentage of functional pancreatic beta cells, and improvement of serum glucose levels, following vitamin K2 intervention. This information correlates with the final results of our study that demonstrated a decrease in serum levels of undercarboxylated osteocalcin following the intervention phase, supporting the hypothesis that an increase in vitamin K2 will in fact also increase the carboxylation of osteocalcin and reducing undercarboxylated osteocalcin [31]. This phenomenon can be observed in the current study given that there was a significant reduction of this molecule at the end of the intervention phase when compared to the beginning.

Similarly, an increased insulin sensitivity following vitamin K2 administration has also been described, thus increasing its utilization by cells and decreasing its concentration, just as was reported in the statistical analysis (Table 4) [30].

The potential benefits of vitamin K supplementation are related to its effect on protein carboxylation which in turn are related to glucose metabolism (e.g. osteocalcin). Increasing adiponectin that is previously reported the insulin-sensitizing effect and the decrease of inflammation by NF-kB regulation. The effects of Vitamin K over glucose metabolism are under debate for multiple considerations; it is not clear which isoform (K1 or K2) has the highest effect on improving glucose metabolism and decreasing the insulin resistance. The difference in the follow-up trials, from weeks to years and dosage are factors to keep in mind when discussing vitamin K supplementation effects. Thus, the underlying mechanisms of Vitamin K on insulin sensitivity or glucose metabolism remain unclear [21].

The secretion of insulin depends on the availability of calcium and adequate concentrations of vitamin D. For this reason, vitamin D deficiency would lead to deterioration in the use of glucose and it is also for this reason that supplementation with this vitamin contributes to an improvement in insulin sensitivity, leading to a reduction in circulating glucose levels, as was observed in our study. This would indicate that supplementation with vitamin D could be considered as a possible adjunctive therapy in the integral management of T2DM, especially considering that all 3 groups demonstrated improvements of HOMA-IR, percentage of functional pancreatic beta cells and glucose levels; all groups reaching serum glucose concentrations below 200 mg/dL. These changes would certainly decrease comorbidities and mortality among this group of individuals while also improving quality of life [25].

After 3 months of treatment, an increase in the cOC concentration was observed in all studied groups, however, only in the total population and in the group with vitamin K2 supplementation was statistically significant, which could be explained by the action of vitamin D3 and K2 on the mineral deposit in the bone and by the action of vitamin K2 as a coenzyme of carboxylases during carboxylation of osteocalcin.

Vitamin D3 administration significantly decrease the concentration of uOC, at the end of treatment. It should be remembered that bone resorption is the main mechanism of release of uOC from the bone, which is produced by the acidic pH generated by the acid hydrolases of the osteoclasts, then apparently the therapeutic doses of the Vitamin D3 decrease bone resorption and therefore the release of uOC [32–34].

Taken together, the individual and synergic effects of vitamins D and K could increase insulin sensitivity, osteocalcin carboxylation, and improve overall bone and glucose homeostasis. According to Diaz Curiel, the metabolic effects of vitamin D3 supplementation could be more prominent in populations with significant vitamin D deficits [35], therefore the use of vitamin D3 alone or in combination with vitamin K2 could be a good therapeutic strategy to simultaneously improve glucose homeostasis and bone health, which is greatly affected in patients with diabetes.

Reports have indicated that following vitamin D supplementation there may be changes, such as an initial increase, in the concentration of undercarboxylated osteocalcin, however, due to the presence of insulin resistance in the context of T2DM more of this molecule must remain in the carboxylated range to increase insulin sensitivity and thus decrease serum glucose levels as demonstrated in this study.

It is suggested the consideration of uOC/cOC or cOC/uOC indexes and the undercaboxylated to total osteocalcin index (uOC/tOC) more than just OC concentrations alone, as described by different authors [21–23]. The T2DM included patients showed an unexpected lessening in the uOC/cOC index along with improved insulin sensitivity and glucose metabolism. In the present study significant reductions of the uOC/cOC index in total studied population, the vitamin D3 and vitamin D3 + K2 supplementation were observed. However, the average index in all treatment groups is > 1, which does not approximate the risk value described by Villafán-Bernal et al. who stated that a value less than 0.31 is correlated with poor metabolic control of T2DM [23]. Conversely, in a study conducted in Polish individuals, it is proposed an inverse ratio cOC/uOC [22] with no clear interpretation of the presented data. Although, the uOC/cOC index was statistically significant different when compared the before to after treatment in the whole sample (40 subjects), it did not show any relation with the included variables. More studies about the uOC/cOC or cOC/uOC indexes are needed in order to clarify this matter.

Regarding the uOC levels in this study, it is important to consider the administration of antidiabetic agents such as metformin among others. Particularly, metformin was the major antidiabetic agent taken by the included patients. Metformin belongs to the pharmacological family of biguanides and its property to modify the tOC/uOC levels has been mentioned [24]. Metformin has the potential to enhance insulin sensitivity in different tissues via AMPK activation, its effect on osteocalcin induction also through the activation of AMPK has been studied in mouse osteoblastic cell lines and, the benefits of this drug on bone health with T2DM patients suggest its usage to treat T2DM-bone fragility [36].

Another aspect to consider is lifestyle, considering that we found there were two major problems that limited the adequate evolution of the patient and finally increase the mortality rate of this population; the first is excessive alcohol consumption, a practice that despite being conditioned, showed a percentage of 57.5%, above of results reported by Torres et al., 2009 who described an alcohol consumption prevalence of 50%, alcohol consumption produces a secondary resistance to insulin and recurrent hyperglycemias that condition the quality of life of the patient with T2DM [37]. The second would be a sedentary lifestyle, its prevalence has been increasing in all age groups, where patients with T2DM are not the exception. It has been reported 33.8% of the general population had a sedentary lifestyle [38]. This unfortunately increases the likelihood of general complications associated with T2DM as well as increasing the risks posed by other chronic conditions such as obesity, hypertension, and cardiovascular disease. Our study observed that around 60% of participants did little to no exercise, which should definitely be a critical point for discussing future health strategies with the hopes of decreasing complications associated with T2DM [39].

The consumption of tobacco among diabetic patients represents another important factor. Despite the many pulmonary complications that can arise. A large percentage of diabetic patients actively smoke and according to López Zubizarreta et al., 2017 around 23.7% of the general population have T2DM and also consume tobacco, these results were very similar to the 17.5% observed in the current study, where we observed that alcohol consumption was actually more prevalent than tobacco use among the studied population [38].

Some studies, like Castro et al., 2015, associate the pathophysiological mechanism involved in the increase of systolic blood pressure, like alterations in calcium homeostasis, with vitamin D3 deficiency, meaning that an inadequate amount of this molecule among the general population, coupled with the presence of T2DM, could cause a significant rise in blood pressure. Therefore, the use of vitamin D supplements to reduce the risk or progression of arterial hypertension could be an adequate alternative therapeutic option. In fact, within the findings of our investigation, we observed a decrease in both systolic and diastolic blood pressure levels after vitamin D supplementation, which demonstrates the hypothesis described in the literature regarding the cardiovascular benefits of vitamin D consumption [40].

We argue that diverse parameters interplay for the reduction of glucose in the included patients for this research. The vitamin supplementation K and D stimulate glucose metabolism and also induces the uOC which is associated with a decrease in glucose levels. Among different targets of OC is the recent AMPK master glucose sensor that has been associated with this action [37].

Finally, our results should be taken with caution, given that they are limited by some factors, such as sample size, time of follow-up and insulin homeostasis phenotype assessment by a single fasting measure. Some nutritional variables related to vitamin K and D supplementation, were not considered. Such as Serum vitamin K and D determinations before and after treatment as well as the influence of nutritional consumption (diet) to assess the appropriate intake of the vitamins. Also, a possible limitation is the employed methodology, a widely immunoassay for uOC determination, which has been described for some authors as inaccurate for its purpose with the possibility to lead an overestimation of uOC levels [19, 41]. It is important to mention that the manufacturer does not express that information in the technical sheet.

## Conclusions

Individual or combined supplementation with vitamins D3 and K2 significantly decreases the glucose levels and % of functional pancreatic beta cells, while D3 and D3 + K2 treatments also induce a reduction in the uOC/cOC index. Only in the group with vitamin D3 supplementation, it was observed a reduction in undercarboxylated osteocalcin while vitamin K2 increased the carboxylated osteocalcin levels.

## Data Availability

Data on the intervention performed on the participants used to support the findings of this study are included in the article. The data used to support the findings of this study can be disclosed by request to the Research, Bioethics and Biosafety Committee of the University Center of Health Sciences of the University of Guadalajara, with whom you can contact MD, Ph.D. Barbara Vizmanos Lamotte. cinv.cucs.udg@gmail.com.

## Abbreviations

T2DM: Type 2 diabetes mellitus
cOC: Carboxylated osteocalcin
uOC: Undercarboxylated osteocalcin
DM: Diabetes mellitus
VD3: Vitamin D3
VK2: Vitamin K2
HOMA-IR: Homeostatic model assessment-insulin resistance
SBP: Systolic blood pressure
DBP: Diastolic blood pressure
FPβC: Functional pancreatic β cells
VLDL: Very low density lipoprotein
BMI: Body mass index
% FPβC: Percentage of functional pancreatic beta cells
ELISA: Enzyme-linked immunoSorbent assay
